# Liquid-liquid phase separation as a major mechanism of plant abiotic stress sensing and responses

**DOI:** 10.1007/s44154-023-00141-x

**Published:** 2023-12-11

**Authors:** Xin Liu, Jian-Kang Zhu, Chunzhao Zhao

**Affiliations:** 1 Shanghai Center for Plant Stress Biology, CAS Center for Excellence in Molecular Plant Sciences, Chinese Academy of Sciences, Shanghai, China; 2 Institute of Advanced Biotechnology and School of Life Sciences, Southern University of Science and Technology, Shenzhen, China; 3 Center for Advanced Bioindustry Technologies, Chinese Academy of Agricultural Sciences, Beijing, China

**Keywords:** Liquid-liquid phase separation, Biomolecular condensates, Stress sensors, Osmotic stress, High temperature

## Abstract

Identification of environmental stress sensors is one of the most important research topics in plant abiotic stress research. Traditional strategies to identify stress sensors or early signaling components based on the cell membrane as a primary site of sensing and calcium signal as a second messenger have had only limited successes. Therefore, the current theoretical framework underlying stress sensing in plants should be reconsidered and additional mechanisms need to be introduced. Recently, accumulating evidence has emerged to suggest that liquid-liquid phase separation (LLPS) is a major mechanism for environmental stress sensing and response in plants. In this review, we briefly introduce LLPS regarding its concept, compositions, and dynamics, and then summarize recent progress of LLPS research in plants, emphasizing the contribution of LLPS to the sensing of various environmental stresses, such as dehydration, osmotic stress, and low and high temperatures. Finally, we propose strategies to identify key proteins that sense and respond to environmental stimuli on the basis of LLPS, and discuss the research directions of LLPS in plant abiotic stress responses and its potential application in enhancing stress tolerance in crops.

## Introduction

In the natural environments, plants often suffer a variety of adverse environmental stresses, such as high salinity, drought, low and high temperatures, any of which can become a major constraint on plant growth and even cause plant death (Zhu 2016). In order to adapt to these unfavorable conditions, plants need to be capable of perceiving and transducing stress signals to adjust growth and reprogram gene expression and metabolism to achieve a new balance between growth and stress tolerance. Understanding the molecular mechanisms of stress responses in plants will be helpful to guide us to breed and cultivate stress resilient crops, and thus contribute to global food security and the sustainable development of agriculture.

In the last several decades, the molecular mechanisms underlying the responses of plants to different environmental stimuli have been extensively studied. However, the majority of studies focused on intracellular signal transduction pathways, and the initial sensing of various stresses was rarely investigated. One of the challenges of identifying stress sensors is that it is difficult to understand how the physical properties of environmental stresses are converted to chemical signals that are commonly studied in plants. It has been well known that almost all environmental stresses can trigger the influx of calcium into the cytosol, which usually occurs in few seconds (Zhu et al. 2013). Therefore, the immediate and transient rise of cytosolic calcium was widely used as a marker to identify the components that are responsible for the early perception of abiotic stress in plants. Using this strategy, OSCA1 (reduced hyperosmolality-induced calcium increase 1) and MOCA1 (monocation-induced [Ca^2+^] increases 1) were identified and reported as osmotic and salt stress sensors, respectively (Jiang et al. 2019; Yuan et al. 2014). *OSCA1* encodes a plasma membrane-localized protein that is required for rapid calcium influx under osmotic stress (Yuan et al. 2014), and the function of OSCA1 protein as a calcium channel is supported by a structural study (Liu et al. 2018). *MOCA1* encodes a Golgi-localized glucuronosyltransferase that is required for the biosynthesis of GIPC (glycosyl inositol phosphorylceramide) sphingolipids, an integral part of the plasma membrane. In *moca1* mutants, GIPCs are obviously reduced, leading to a deficiency in salt-triggered Ca^2+^ spikes. The conclusion that GIPCs function in salt sensing is based on data showing that GIPCs can directly bind to Na^+^. Although GIPCs are considered as a salt sensor, the calcium channel mediating the GIPCs-regulated Ca^2+^ influx under salt stress is still unknown (Jiang et al. 2019). In rice, COLD1 was reported to serve as a cold stress sensor, which is supported by its role in the regulation of Ca^2+^ influx under low temperature conditions (Ma et al. 2015). The calcium channel regulated by COLD1 to control Ca^2+^ influx also remains elusive.

Although several abiotic stress sensors have been discovered based on Ca^2+^ signaling, the identification of the Ca^2+^ channels involved and how the channels are regulated by the supposed sensors is challenging. Importantly, disruptions of these stress sensors usually only partially affect the physiological responses of plants to stress (Jiang et al. 2019; Yuan et al. 2014). It has been proposed that plants may sense environmental stresses not only at the plasma membrane, but also in the cell wall and different intracellular organelles or compartments (Zhu 2016). It is highly likely that various additional mechanisms must exist in plants to perceive various environmental signals. Recently, formation of biomolecular condensates driven by liquid-liquid phase separation (LLPS) has been proposed as a mechanism for organisms to sense external signals. In Arabidopsis, EARLY FLOWERING 3 (ELF3) and phytochrome B (phyB) function as thermosensors via the behavior of LLPS (Jung et al. 2016; Jung et al. 2020; Legris et al. 2016), and SEUSS (SEU) is considered as a potential hyperosmotic stress sensor because of its ability to form nuclear condensates upon exposure to osmotic stress (Wang et al. 2022). These pioneering studies provided important paradigms to show that LLPS is critical for plants to sense environmental stresses. In this review, we introduce the basic property of LLPS and examples of LLPS-driven biomolecular condensates in plants, and then focus on the elucidation of the roles of LLPS in abiotic stress responses in plants, particularly its contribution to stress sensing. For a comprehensive introduction of the roles of LLPS in other plant biological processes, such as plant growth and development, as well as plant immunity, several excellent reviews can be referred (Emenecker et al. 2020, 2021; Wang et al. 2023; Xu et al. 2021).

## Concept of liquid-liquid phase separation

**Fig. 1:**
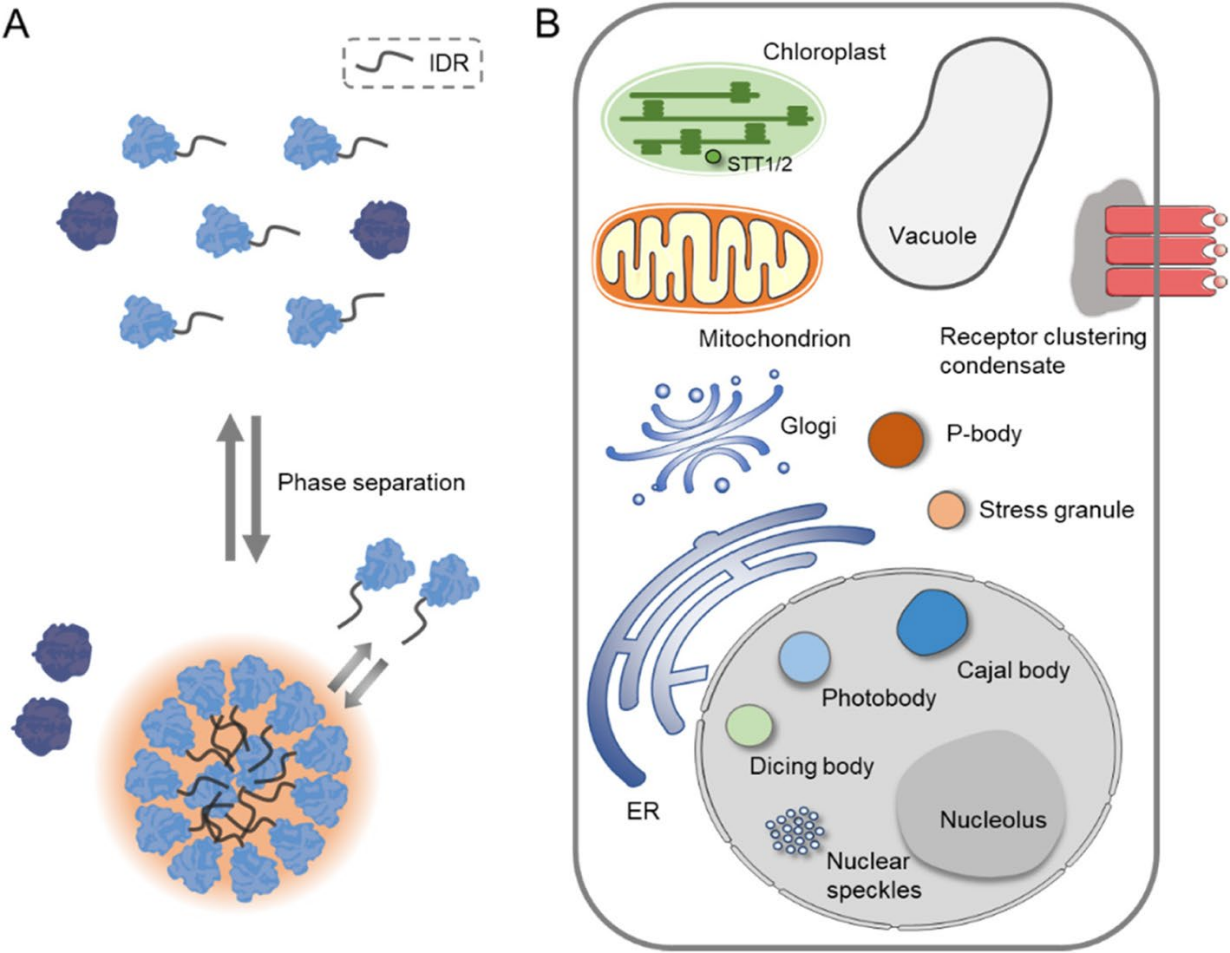
Phase separation-triggered biomolecular condensates in plants. **A** A simple schematic diagram showing the formation of condensed membraneless droplets driven by the phase separation proteins that harbor intrinsically disordered regions (IDRs). **B** The representative types of biomolecular condensates in plants are shown, including the nucleolus, Cajal bodies, photobodies, dicing bodies, and nuclear speckles that occur in the nucleus, and stress granules and P-bodies that are observed in the cytosol. Phase separation-triggered biomolecular condensates have also been observed in chloroplasts and at the vicinity of cell surface

The concept of LLPS was first proposed in 2009 by Brangwynne and colleagues, describing a phenomenon that P granules in the germ cells of *Caenorhabditis elegans* are rapidly dissolved and condensed, which is reminiscent of the phenomenon of phase transition (Brangwynne et al. 2009). Later this concept was widely exploited to explain the behaviors of membraneless compartments that ubiquitously exist in cells. LLPS, as a physical chemistry concept, describes the phenomenon that under certain conditions initially homogeneously distributed components separate from a diluted phase into a more condensed phase, and this phase is characterized by the hallmarks of sphericity, fluidity, and reversibility (Hyman et al. 2014). LLPS-driven biomolecular condensates are composed of various macromolecules, such as proteins, nucleic acids, and lipids, and compartmentation of these macromolecules in different condensates enables the spatiotemporal regulation of a variety of cellular activities, such as signal transduction, cargo sorting, protein localization, RNA transcription, and protein translation (Tsang et al. 2020). Proteins that undergo phase separation usually contain one or more intrinsically disordered regions (IDRs), also known as low complexity domains (LCDs), which are commonly abundant in polar, aromatic, and/or charged amino acids and exhibit a significant degree of conformational flexibility (Das et al. 2015). In general, IDRs in the phase-separation proteins are important for driving the formation of biomolecular condensates, because these disordered regions provide surfaces for the multivalent weak interactions with other components (Dignon et al. 2020) (Fig. 1A). However, it should be clarified that the proteins harboring IDRs do not necessarily have the capacity to form condensates, and the molecular condensates observed in cells are not necessarily formed via LLPS.

## Liquid-liquid phase separation in plants

The biomolecular condensate in plant cell was first reported in tomato in 1983, and in this study cytoplasmic heat shock granules were observed in tomato cell cultures and leaves after being subjected to heat shock (Nover et al. 1983). Since then, many different types of biomolecular condensates in plants have been discovered, and these condensates include the nucleolus, Cajal bodies, photobody, dicing body, and nuclear speckles that occur in the nucleus, and stress granules (SGs) and P-bodies (PBs) that are usually observed in the cytosol (Emenecker et al. 2020) (Fig. 1B). As an essential subcellular structure in the nucleus, the nucleolus is an influential factory for rRNA (ribosomal RNA) synthesis and ribosome biogenesis (Kalinina et al. 2018). Recent findings suggest that the nucleolus also plays a crucial role in the regulation of telomerase activity and gene silencing in response to biotic and abiotic stresses. STRESS RESPONSE SUPPRESSOR1 (STRS), a protein localized in the nucleolus, is rapidly translocated into the nucleoplasm in response to salt or heat stress. Arabidopsis *strs* mutant exhibits enhanced tolerance to salt, osmotic and heat stress (Kant et al. 2007). Cajal bodies, acting as subnuclear compartments formed through LLPS, are required for RNA metabolism and small nuclear ribonucleoprotein (snRNP) biogenesis (Morris 2008). In addition, Cajal bodies act as centers for the assembly of AGO4/NRPD1b/siRNA complex, facilitating its function in RNA-directed DNA methylation at target loci (Li et al. 2006; Wang et al. 2020). Photobody is a subnuclear compartment that is formed by phytochromes in response to light irradiation. In the photobody, phytochromes mediate the degradation of growth-promoting factor PIFs, leading to the transition of plants from skotomorphogenesis to photomorphogenesis. The photobody assembly is a reversible process, as either far-red light or darkness can trigger the dissociation of photobody (Al-Sady et al. 2006; Kim et al. 2023; Kircher et al. 2002; Wang et al. 2021; Yamaguchi et al. 1999). Dicing bodies are plant-specific subnuclear condensates that regulate RNA silencing (Liu et al. 2012). miRNAs are produced via the cleavage of pri-miRNAs by a complex composed of DICER-LIKE 1 (DCL1), SERRATE (SE), and HYPONASTIC LEAVES 1 (HYL1), and these three components are localized in the nuclear dicing bodies in a manner of phase separation (Li et al. 2021; Xie et al. 2020).

Multiple nuclear speckles have been observed in the nucleus, but currently the exact roles of these nuclear punctures are still largely unknown. In mammalian cells, nuclear speckles are involved in the regulation of RNA processing, cell differentiation, immunity, and metabolic alterations (Lamond and Spector 2003). In plants, nuclear speckles have been implicated in the partitioning of important regulatory factors, such as RNAs or proteins, into discrete subnuclear domains, the process of which is important for the control of the amount of these components in the nucleoplasm and ultimately fine-tunes downstream gene expression (Spector and Lamond 2011). Recent studies indicate that multiple nuclear speckles-localized proteins play important roles in regulating plant responses to both internal and external cues (Koroleva et al. 2009; Na et al. 2015; Park et al. 2020). For example, eukaryotic initiation factor 4A-III (eIF4A-III), a putative anchor protein in Arabidopsis, is localized in the nucleoplasm during normal growth, but migrates to the nucleolus and splicing speckles in response to hypoxia (Koroleva et al. 2009). In another example, the RNA-binding protein UBP1-associated protein 2 (UBA2) is localized in the nuclear speckles and participates in the regulation of leaf senescence (Na et al. 2015). In rice, immunophilin family protein OsFKBP20-1b interacts with the splicing factor OsSR45 in both nuclear speckles and cytoplasmic foci to regulate the transcription and pre-mRNA splicing of stress-responsive genes under abiotic stress conditions (Park et al. 2020). Recently, a study revealed that, in response to DNA damage, the plant-specific histone methyltransferase SUVR2 forms condensates at the damage sites, and thus promotes the compaction of chromatin structure through H3K9 methylation modification (Liu et al. 2022).

SGs and PBs are cytoplasmic membraneless condensates that are predominantly required for the post-transcriptional and translational regulation of gene expression (Youn et al. 2019), and a growing body of evidence has revealed that both compartments are critical for stress tolerance in plants (Solis-Miranda et al. 2023). One of the typical features of SGs and PBs is that both compartments are composed primarily of proteins and RNAs that are assembled via LLPS (Youn et al. 2019), and most of the compartmentalized proteins harbor IDRs (Solis-Miranda et al. 2023). Although SGs and PBs presumably exhibit similar properties and frequently communicate with each other (Yan et al. 2022), they generally exhibit distinct functions in RNA metabolism. SGs are capable of sequestering mRNAs that are stalled in translation initiation, and temporarily store mRNAs during stress conditions, the action of which allows for expeditious revival of gene translation once stress is relieved (Maruri-López et al. 2021). In Arabidopsis, several core components of SGs have been identified, including the RNA-binding protein 47b (Rbp47b), oligouridylate binding protein 1 (UBP1), eukaryotic initiation factor (eIF4E1), tandem zinc finger 3 (TZF3), tudor staphylococcal nuclease (TSN), and poly(A) binding proteins, and these core components are essential for the initial assembly of SGs (Chantarachot and Bailey-Serres 2018). A recent study reported that Rbp47b functions as a sensor of phenolic acids (PAs) in Arabidopsis to trigger SG formation and global translation inhibition, and finally mediates interspecific competition (Xie et al. 2023). PBs are characterized by the existence of DECAPPING (DCP) proteins that are predominantly involved in the degradation of mRNAs (Baumberger and Baulcombe 2005; Xu et al. 2006). Another difference between these two membraneless organelles is that SGs are usually only detected under stress conditions, while PBs constitutively exist in cells (Zhu et al. 2022).

In the past few years, studies of the biological roles of phase separation in Arabidopsis have led to important discoveries. In chloroplasts, two ankyrin proteins, STT1 and STT2, are required for sorting cpTat pathway proteins and targeting these substrates to thylakoid membrane via the formation of condensed droplets (Ouyang et al. 2020). In Arabidopsis, the coiled-coil protein FLL2 promotes the prion-like domain-containing protein FCA to form nuclear bodies via LLPS, and these biomolecular condensates are capable of compartmentalizing 3′-end processing factors to enhance polyadenylation at specific sites, and resulting in flowering time regulation (Fang et al. 2019). In another example, photo-excited cryptochrome 2 (CRY2) forms nuclear speckles via LLPS, which subsequently recruit m^6^A writer complex to the condensates to modulate the m^6^A methylation of mRNAs associated with circadian rhythms (Wang et al. 2021). In tomato, it was reported that H_2_O_2_ in plant shoot apical meristem (SAM) triggers the phase separation of TERMINATING FLOWER (TMF) via an oxidative modification, and the condensation of TMF promotes its binding to the promoter of a floral identity gene *ANANTHA* to repress its expression (Huang et al. 2021).

## Roles of LLPS in abiotic stress sensing in plants

In addition to plant development and immunity, several examples of LLPS have been reported in abiotic stress responses in plants. One of the important features of LLPS is that the dynamics of phase separation is influenced by the physicochemical properties of solvent, such as ion concentration, temperature, pH, and redox state, and these factors are changeable under environmental stresses (Dignon et al. 2020). Therefore, it is logical to assume that the perturbations of these ﻿physical factors under abiotic stresses can be sensed by certain phase-separation proteins in plants. Indeed, the involvement of LLPS in sensing environmental stimuli, such as hydration, osmotic stress, and low and high temperatures, has been reported recently, which suggest a broad mechanism of plant sensing of environmental stress.

**Fig. 2:**
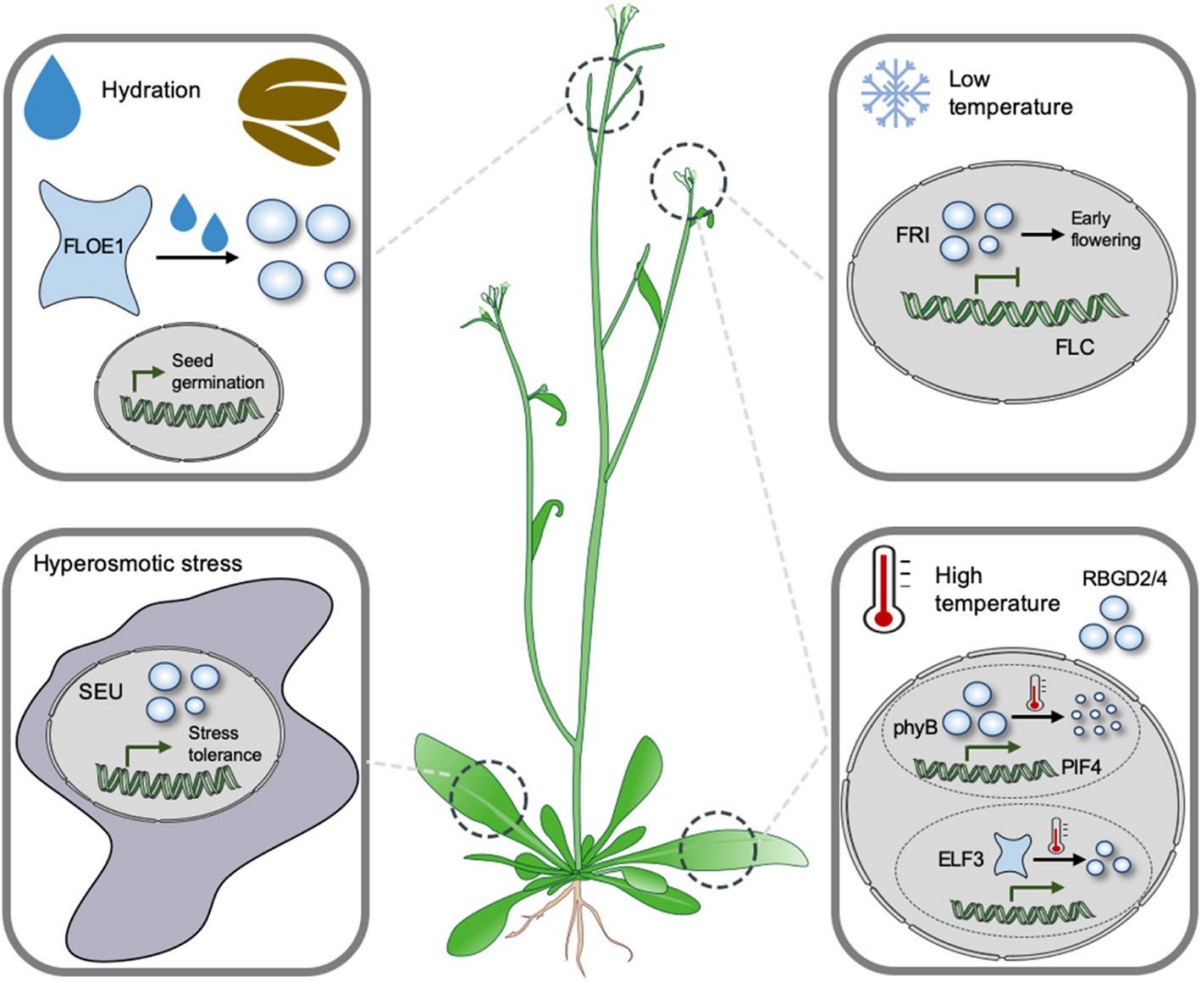
Illustration of the roles of LLPS in the sensing of environmental stimuli in plants. FLOE1 forms condensates under hydration conditions, and thus promotes seed germination. SEU is required for the sensing of osmotic stress-triggered mocelular crowding via the formation of LLPS in the nucleus. Low temperatures enhance the condensation of FRI protein, which in turn regulates flowering via the inhibition of *FLC* expression. PhyB and ELF3 are both considered as direct thermosensors, which are involved in the regulation of plant morphology and flowering time under high temperatures, respectively. RBGD2/4 are localized in stress granules and paritiplate in the regulation of mRNA metabolism under heat stress

Seed germination is the first step in the life cycle of an individual plant, and thus it needs to be tightly regulated to avoid the growth of plants under unfavorable conditions, such as high salinity and drought stress, both of which can cause hyperosmotic stress. Seeds prefer to delay their germination when grown in the soils with hyperosmotic stress conditions, but how plants perceive water potential to control seed germination is still unknown. Recently, Dorone et al. identified a prion-like protein, FLOE1, that undergoes hydration-dependent phase separation and acts as a sensor of water potential to regulate seed germination (Dorone et al. 2021) (Fig. 2). At the seed germination stage, when dormant seeds are immersed in water, FLOE1 rapidly forms cytoplasmic condensates via LLPS, which is a critical step to promote seed germination. However, when the seeds are immersed in solution with high salinity or glycerin that causes dehydration, no cytoplasmic condensates are observed. The condensation of FLOE1 is a reversible process, as the hydration-induced condensation of FLOE1 disappears when the water is changed to high salts or glycerin (Dorone et al. 2021). The germination rate of *floe1-1* mutant is similar to the wild type under hydration conditions. However, under water deprivation conditions induced by high salinity or mannitol, *floe1-1* mutant germinates faster than the wild type, indicating that FLOE1 is required for the inhibition of seed germination under dehydration conditions. Currently the molecular mechanism underlying the FLOE1-meidated regulation of seed germination is still largely unknown. It has been well known that ABA signaling plays a key role in the control of seed germination, especially under stress conditions (Chen et al. 2020). Whether the formation of FLOE1 condensate after seed imbibition is a key event to attenuate ABA signaling is worthy of further investigation. In addition, whether FLOE1 directly senses water potential and whether other components also participate in the formation of condensates after seed imbibition need to be further studied. As described above, stress granule and P-body mainly exist in the cytosol, so whether the cytoplasmic condensate of FLOE1 processes the features of these two membraneless compartments also needs to be further analyzed.

Osmotic stress caused by high salinity or drought stress in natural environments not only reduces water potential, but also leads to intracellular molecular crowding. Wang et al. reported that transcriptional regulator SEU forms nuclear condensates under osmotic stress, which is likely caused by the dehydration-triggered molecular crowding in the nucleus (Wang et al. 2022) (Fig. 2). SEU protein harbors two α-helical structures in the N-terminal IDR, and in response to osmotic stress-triggered molecular crowding, these two α-helical structures are conformationally changed to a more condensed state, the behavior of which is required for the formation of biomolecular condensates. SEU protein lacking two α-helical domains can no longer form condensates under osmotic stress. Phenotypic analysis revealed that *seu-6* mutant displays an increased sensitivity to osmotic stress, and transcriptional profiling showed that SEU is required for the expression of osmotic stress-responsive genes. Taken together, these results support a scenario in which SEU positively regulates plant tolerance to osmotic stress via crowding-triggered LLPS (Wang et al. 2022). This study also relays a message that intracellular molecular crowding is probably one of the internal signals that are utilized by plants to sense environmental stresses. Although experimental evidence has supported the conformational changes of the α-helical domains after macromolecular crowding, how their structural changes drive the formation of SEU nuclear condensates is still elusive, and the accurate biological function of SEU in the condensates is also still unknown. During our study, we noted that α-helical domains are commonly identified in the IDRs of many intrinsically disordered proteins (IDPs), so whether α-helical domain has a special function in assisting the formation of biomolecular condensates needs to be studied in more detail.

Low temperature is not only an abiotic stress that adversely affects plant growth, but also acts as an environmental signal for the regulation of flowering time. In Arabidopsis, FRIGIDA (FRI)-dependent activation of FLC plays a key role in the regulation of flowering time (Choi et al. 2011), but how the function of FRI is regulated in response to fluctuating temperatures is still largely unknown. Zhu et al. found that FRI-GFP forms nuclear condensates, and the size and number of condensates are increased after cold treatment (Zhu et al. 2021) (Fig. 2). Further analysis indicated that the FRI-interacting protein FRL1 is required for the cold-enhanced condensation of FRI-GFP in the nucleus. FRI protein harbors a C-terminal IDR, and deletion of this domain abolishes the condensation of FRI protein under low temperatures. The biological significance of this nuclear condensates formation is to prevent the access of FRI protein to the *FLC* locus, resulting in a decreased expression of *FLC* gene. Formation of FRI condensates upon cold exposure is a reversible process, as the condensates disappear within 5 h after being returned to warm conditions (Zhu et al. 2021). These results may suggest that FRI or its interacting proteins are capable of directly sensing low temperatures. Currently, the involvement of LLPS in the regulation of cold acclimation and freezing tolerance has not been reported. Considering that plants induce early cold stress responses via transcriptional cascades (Zhao et al. 2015), we speculate that LLPS is most likely involved in the regulation of the cold-responsive gene expressions. In future, the IDPs that are required for cold stress response need to be identified and functionally studied.

High temperature is another environmental factor that considerably affects plant growth and yield, as well as the geographical distribution of plants. For Arabidopsis, high temperature can be divided into high ambient temperature that refers to temperatures ranging from 27 °C to 32 °C, and heat stress that refers to temperatures above 37 °C (Vu et al. 2019). In 2016, two parallel studies reported that phytochrome B (phyB) acts as a thermosensor in Arabidopsis (Jung et al. 2016; Legris et al. 2016). phyB is a red/far-red light receptor that is activated upon red light irradiation by a mean of the conversion from the inactive Pr form to active Pfr form. The active Pfr form is transported to the nucleus to form photobodies (Bae and Choi 2008). In contrast to red light stimulus, high ambient temperature promotes the reversion of phyB from Pfr to Pr, leading to the reduction of the size and number of photobodies in the nucleus, and finally promoting the expression of thermomorphogenesis-related genes (Jung et al. 2016; Legris et al. 2016) (Fig. 2). In 2019, another group reported that phyB also plays an important role in temperature sensing during the daytime. The transcriptional activator HEMERA interacts directly with PIF4 to promote high ambient temperature-dependent PIF4 accumulation and the induction of temperature-responsive genes (Qiu et al. 2019). The LLPS property of photobodies was not well characterized until a recent study reporting that phyB condensation in the nucleus is required for its function as a thermosensor (Chen et al. 2022). The work showed that the intrinsically disordered N-terminal extension of phyB is responsible for directly sensing temperature changes and for regulating photobody formation via LLPS (Chen et al. 2022). *ELF3* encodes a plant-specific nuclear protein, which together with ELF4 and LUX ARRYTHMO (LUX), functions as a circadian rhythm-dependent temperature-responsive transcriptional repressor (Nusinow et al. 2011). Recently, it was shown that ELF3 forms nuclear speckles through LLPS in response to changes in ambient temperature to regulate flowering (Jung et al. 2020) (Fig. 2). ELF3 contains a polyQ (polyglutamine) repeat, which is embedded within a predicted prion domain (PrD). Intriguingly, the length of polyQ repeat correlates with thermal responsiveness, and plants living in geographically high temperature areas have a longer polyQ repeat. ELF3 assembles into nuclear speckles within minutes in response to high temperatures and this condensation is a reversible process that depends on the PrD motif. Sequestration of ELF3 protein into nuclear speckles relieves its transcriptional repression of targeted genes and thus promotes hypocotyl elongation (Jung et al. 2020). In another study, Zhu et al. found that the RNA-binding glycine-rich D2 and D4 (RBGD2/RBGD4) proteins are required for heat stress resistance in Arabidopsis (Fig. 2). *rbgd2-1 rbgd4-1* double mutant plants display a lower survival rate compared with wild-type plants under heat stress. RBGD2/4 are condensed into SGs upon heat treatment, and the tyrosine residue array localized in the IDR of these two proteins is essential for driving their condensation in SGs. The condensation of RBGD2/4 into SGs allows RBGD2/4 to regulate the metabolism of heat-responsive mRNAs, and thus improves plant tolerance to heat stress (Zhu et al. 2022). In this study, it was also shown that RBGD2/4 are not required for the initial assembly of SGs under heat stress (Zhu et al. 2022), so which components initiate the formation of SGs in response to heat stress is an intriguing question, and discovery of such components may enable us to identify heat stress sensors. It has been recently reported that, TSN proteins, which act as the core components of SGs, mediate the assembly of SnRK1α in the SGs upon exposure to heat stress (Gutierrez-Beltran et al. 2021), but whether TSN proteins process the capacity to sense heat stress needs more experimental evidence. In another study, PB proteome was performed in the four-week-old rosettes of Arabidopsis with or without heat stress (Liu et al. 2023), which provides valuable candidate proteins in PBs that may sense and respond to heat stress.

## A great potential to identify more stress sensors based on LLPS

As described above, several stress sensors have been identified based on the phenomenon of LLPS. These studies suggest that there is a great potential to discover additional stress sensors in plants by identifying more proteins that undergo phase separation in response to environmental stress. Theoretically, proteins that are capable of forming condensates via LLPS usually harbor IDRs or other regions that confer multivalent interactions (Dignon et al. 2020), and such regions can be computationally predicted by bioinformatic tools such as PLAAC (Lancaster et al. 2014), IUPred2A (Erdos and Dosztanyi 2020), ESpritz (Walsh et al. 2012), PrDOS (Ishida and Kinoshita 2007), D2P2 (Oates et al. 2013), or MobiDB (Piovesan et al. 2018). Therefore, proteome-wide analysis of proteins that harbor IDRs can be conducted in Arabidopsis or other plant species, and the IDR-containing proteins can be candidates for testing their capacity to form biomolecular condensates in response to various environmental cues. Using this strategy, there is a good potential to identify stress sensors or early signaling components that regulate stress responses.

In addition to the computational prediction of IDR-containing proteins, experimental approaches to identify phase-separation proteins have also been applied in plants. Recently, Zhang et al. performed a mass spectrometry analysis to identify proteins with a potential to undergo phase separation by using a small molecule biotinylated isoxazole (b-isox) (Zhang et al. 2023). b-isox is capable of forming microcrystals, which can be a platform to trigger the conversion of LCD from a soluble state to a polymerized fiber-like state, thus providing a chance to identify proteins that harbor LCD in b-isox precipitations (Kato et al. 2012). Using this method, 985 proteins with high-confidence phase separation potentials in Arabidopsis were identified (Zhang et al. 2023). This list includes most of the proteins that have been reported to phase separate in plants, such as ELF3, EMB1579, and FCA (Fang et al. 2019; Jung et al. 2020; Zhang et al. 2020). Because biomolecular condensates in plants often occur in response to environmental stresses, the authors also performed b-isox-based assays under a variety of stress conditions, including osmotic stress, high salinity, heat stress, and oxidative stress. Their data show that, compared with the seedlings without stress treatment, 25% of the identified proteins were specifically found in the seedlings after stress treatment, suggesting that some of the phase-separation proteins are specifically involved in the response to environmental stresses. In addition to Arabidopsis, this method was extended to identify phase-separation proteins in other species, including the green alga *Chlamydomonas reinhardtii*, the moss *Physcomitrella patens*, rice (*Oryza sativa*), maize (*Zea mays*), common wheat (*Triticum aestivum*), Chinese cabbage (*Brassica rapa ssp*. *Pekinensis*), and tomato (*Solanum lycopersicum*). These candidate phase-separation proteins can be a valuable resource to identify proteins that sense environmental stresses in plants.

SGs and PBs have been shown to be crucial for the regulation of stress tolerance in plants (Solis-Miranda et al. 2023). Numerous core components (referred to as scaffolds) of these two compartments have been identified (Chantarachot and Bailey-Serres 2018), but knowledge on the roles of these core components in stress responses is limited. In future, high-throughput phenotypic analysis of the mutants of these core components can be performed, which will provide novel insights into the contributions of SGs and PBs to stress tolerance in plants. Importantly, in addition to the core components, SGs and PBs may recruit client components in response to environmental stress. A scaffold and client model for condensates has been proposed to distinguish the components that are essential for the initial formation of condensates and the components that preferentially localize to condensates (Emenecker et al. 2021). Using transgenic plants expressing GFP-fused Rbp47b protein, a marker of SGs, 118 proteins were co-purified with Rbp47b after heat treatment, and 75% of them, including some protein kinases, metabolic enzymes and lipid-binding proteins, are not classified as the core components of SGs (Kosmacz et al. 2019). Functional studies of these client components may help improve our understanding of how plants specifically respond to each environmental stress, since it is possible that the recruited client proteins in SGs or PBs may function as stress sensors to regulate downstream stress responses.

## Conclusions and perspectives

The biological functions of LLPS and the mechanisms underlying their formation are relatively well characterized in human and yeast, but the study of LLPS in plants has just emerged in the last few years. Considering the importance of LLPS in the regulation of a wide range of biological processes, large efforts are needed to study the functional mechanisms of LLPS in the context of plant biology, which will substantially advance our understanding of basic life activities in plants. In the field of plant abiotic stress, the introduction of LLPS provides a great potential to identify key components that regulate stress tolerance. In the future, more proteins that undergo phase separation under stress conditions need to be identified and functionally characterized. The elucidation of the mode of action of these phase-separation proteins will greatly enrich our understanding of how plants sense and respond to environmental stresses. In addition, the LLPS-driven formation of biomolecular condensates has been implicated in the regulation of mRNA metabolism via the formation of mRNA-ribonucleoprotein complexes (Chantarachot and Bailey-Serres 2018). Using RNA immunoprecipitation (RIP) and high-throughput sequencing, more mRNAs that are post-transcriptionally regulated in response to stress conditions may be identified, which would unravel the potentially novel metabolic landscape of stress-responsive mRNAs in plants. Ca^2+^ is an important second messenger that is widely explored by plants to respond to environmental stresses, and currently little is known about the involvement of Ca^2+^ signaling in LLPS formation. In future, the proteins harboring both IDRs and calcium binding domain can be selected to investigate the relationship between Ca^2+^ signaling and LLPS. Despite the membrane-less property of LLPS, the interplay between membranes and LLPS has recently been raised as an interesting research topic (Agudo-Canalejo et al. 2021; Dragwidge and Van Damme 2023; Hatzianestis et al. 2023; Kusumaatmaja et al. 2021), and elucidation of the mechanism underlying this interaction will help us understand how the membrane-localized receptor-like kinases transduce external signals to specifically activate certain intracellular substrates.

Currently, most of the studies on LLPS in plants were performed in the model plant Arabidopsis, and the knowledge of LLPS in crops is still scarce. As mounting evidence supports that LLPS plays important roles in stress tolerance in plants, the identification of stress-related phase-separation proteins in crops will provide valuable genetic resources for the engineering of stress-tolerant crops. Based on computational predictions and experimental approaches (Zhang et al. 2023), the proteins in crops that potentially undergo LLPS have been identified at a proteome level. In future, high-throughput construction of crop mutants that are deficient in phase-separation proteins can be generated and the phenotypic analysis of these mutants under various stress conditions can be performed. In addition, the condensation of these proteins in response to various environmental stresses can be examined. We believe such work will facilitate the discovery of key components in crops that sense and respond to environmental stresses. Because there are a large number of IDPs in plants, the challenge of this field is how to narrow down the candidate IDPs that are specifically involved in the response to environmental stress. We propose that the data derived from transcriptome, proteome, or phosphoproteome after stress treatment can be explored to search for the IDPs that are able to respond to environmental stresses in transcriptional, translational, or posttranslational level. After discovering the stress-responsive IDPs and demonstrating their capacity to form biomolecular condensates under stress conditions, IP-MS or other protein affinity assays can be performed to identify interacting proteins, which will ultimately enable the establishment of the LLPS-based regulatory network in response to environmental stresses.

Regarding the application of LLPS in improving stress tolerance in crops, several strategies can be explored. One of the typical properties of LLPS is that the formation of condensates is protein abundance-dependent, so overexpression of the LLPS-related genes can confer obvious effects on the condensation of phase-separation proteins, and thus enhance stress responses. It has been known that the IDRs in the phase-separation proteins evolved faster than structural domains (Hatzianestis et al. 2023), and as a result more single nucleotide polymorphisms (SNPs) are identified in the IDRs than the structural domains when comparing homologs in different plant ecotypes or cultivars. Therefore, identification of haplotypes that process a higher capacity of protein condensation will enable us to identify stress-tolerant varieties in crops. Moreover, phase-separation proteins usually harbor both IDRs and structural domains, and IDRs play a predominant role in assembling proteins in the condensates, so constructing chimeric proteins that combine IDRs and other functional domains may provide a great potential to create novel functional proteins that improve the adaptation of crops to environmental stress. Finally, screening for small peptides or chemicals that are able to trigger the condensation of phase-separation proteins is also an important strategy for the application of LLPS.

## Data Availability

Not applicable.

## Abbreviations

LLPS: Liquid-liquid phase separation
OSCA1: Reduced hyperosmolality-induced calcium increase 1
MOCA1: Monocation-induced [Ca^2+^] increases 1
GIPC: Glycosyl inositol phosphorylceramide
ELF3: Early flowering 3
phyB: Phytochrome B
SEU: SEUSS
IDRs: Intrinsically disordered regions
LCDs: Low complexity domains
SGs: Stress granules
PBs: P-bodies
rRNA: ribosomal RNA
STRS: Stress response suppressor 1
snRNP: Small nuclear ribonucleoprotein
DCL1: DICER-LIKE 1
SE: SERRATE
HYL1: HYPONASTIC LEAVES 1
eIF4A-III: Eukaryotic initiation factor 4A-III
UBA2: UBP1-associated protein 2
OsSR45: *Oryza sativa* serine/arginine-rich protein 45
Rbp47b: RNA-binding protein 47b
UBP1: Oligouridylate binding protein 1
eIF4E1: Eukaryotic initiation factor 4E1
TZF3: Tandem zinc finger 3
TSN: Tudor staphylococcal nuclease
Pas: Phenolic acids
DCP: Decapping
cpTat: Chloroplast twin arginine translocation
FLL2: FLX-like 2
FCA: Flowering time control protein A
CRY2: Cryptochrome 2
SAM: Shoot apical meristem
TMF: TERMINATING FLOWER
IDPs: Intrinsically disordered proteins
FRI: FRIGIDA
FLC: Flowering locus C
LUX: LUX ARRYTHMO
PrD: Prion domain
RBGD2/4: RNA-binding glycine-rich D2/4
b-isox: Biotinylated isoxazole
RIP: RNA immunoprecipitation
SNPs: Single nucleotide polymorphisms
